# Peroxisomal Acyl-CoA Oxidase Type 1: Anti-Inflammatory and Anti-Aging Properties with a Special Emphasis on Studies with LPS and Argan Oil as a Model Transposable to Aging

**DOI:** 10.1155/2018/6986984

**Published:** 2018-03-25

**Authors:** Joseph Vamecq, Pierre Andreoletti, Riad El Kebbaj, Fatima-Ezzahra Saih, Norbert Latruffe, M' Hammed Saïd El Kebbaj, Gérard Lizard, Boubker Nasser, Mustapha Cherkaoui-Malki

**Affiliations:** ^1^INSERM, HMNO, CBP, CHRU and RADEME EA 7364, Faculté de Médecine, Université Lille 2, Lille, France; ^2^Laboratoire BioPeroxIL (Biochimie du Peroxysome, Inflammation et Métabolisme Lipidique) EA 7270/Inserm, Université Bourgogne-Franche Comté, Dijon, France; ^3^Laboratoire des Sciences et Technologies de la Santé, Institut Supérieur des Sciences de la Santé, Université Hassan Premier, Settat, Morocco; ^4^Laboratoire de Biochimie et Neurosciences, Faculté des Sciences et Techniques, Université Hassan I, Settat, Morocco; ^5^Laboratoire de recherche sur les lipoprotéines et l'Athérosclérose, Faculté des Sciences Ben M'sik, Université Hassan II-Mohammedia-Casablanca, Casablanca, Morocco

## Abstract

To clarify appropriateness of current claims for health and wellness virtues of argan oil, studies were conducted in inflammatory states. LPS induces inflammation with reduction of PGC1-*α* signaling and energy metabolism. Argan oil protected the liver against LPS toxicity and interestingly enough preservation of peroxisomal acyl-CoA oxidase type 1 (ACOX1) activity against depression by LPS. This model of LPS-driven toxicity circumvented by argan oil along with a key anti-inflammatory role attributed to ACOX1 has been here transposed to model aging. This view is consistent with known physiological role of ACOX1 in yielding precursors of specialized proresolving mediators (SPM) and with characteristics of aging and related disorders including reduced PGC1-*α* function and improvement by strategies rising ACOX1 (via hormonal gut FGF19 and nordihydroguaiaretic acid in metabolic syndrome and diabetes conditions) and SPM (neurodegenerative disorders, atherosclerosis, and stroke). Delay of aging to resolve inflammation results from altered production of SPM, SPM improving most aging disorders. The strategic metabolic place of ACOX1, upstream of SPM biosynthesis, along with ability of ACOX1 preservation/induction and SPM to improve aging-related disorders and known association of aging with drop in ACOX1 and SPM, all converge to conclude that ACOX1 represents a previously unsuspected and currently emerging antiaging protein.

## 1. Introduction

Vegetal compounds and oils are current trends for active research. One reason lies in the consideration that nature forms a whole and may provide living beings with both pathogenic and curative factors. Beside vegetal poisons [1], some vegetal compounds as for instance those extracted from *Digitalis purpurea* and *Camptotheca acuminata* tree may be therapeutically active [2, 3] or in turn may serve as guide compounds for medicinal chemistry design in cardiovascular disorders and cancer. It remains currently unclear whether, however, such real curative medical indications may be attributed as a general rule to the bulk of vegetal-derived compounds or oils endowed with health-improving properties. In this respect, argan oil is currently becoming largely acclaimed for a large variety of health and wellness virtues [4, 5]. The anti-inflammatory potential of argan oil has been recently addressed by evaluating its effects towards the highly inflammatory LPS using a murine model of sepsis [6]. In this disorder, peroxisomal Acyl-CoA oxidase 1 (ACOX1) was preserved by argan oil against inactivation by LPS in the same time as the oil protected the liver against most of LPS-driven toxicity and metabolic homeostasis disruption [6]. On the other hand, ACOX1 initiates the peroxisomal pathway ensuring retroconversion of polyunsaturated fatty acids [7]. Key role of ACOX1 in biosyntheses of eicosanoids (E series) and docosanoids (D series) further yielding specialized proresolving mediators (SPM) (resolvins, neuroprotectins, and maresins) [8, 9] is stressed along with preserved or increased ACOX1 activity in models of diabetes and metabolic syndromes. The alterations in SPM production and their roles as potential therapeutic candidates in many other pathological states linked to aging such as neurodegenerative disorders (e.g., Alzheimer's disease), macular degeneration, and vascular disorders (stroke, atherosclerosis, and mitochondrial dysfunction in senescent brain) are further developed.

Olive oil contains about 70% oleic acid while argan oil harbors 45% oleic acid and 35% linoleic acid, indicating that argan oil is richer in polyunsaturated fatty acids [10]. Early studies on argan oil revealed its beneficial effects on lipid metabolism and antioxidant status through drops in plasma low density lipoprotein-cholesterol (LDL-cholesterol) and lipoperoxides, along with a rise in plasma tocopherol concentration [11]. In this respect, argan oil contains a high level of antioxidant compounds including gamma-tocopherol and also ferulic acid which exhibit ROS-scavenging and anti-inflammatory properties [12]. Cumulated activities of these individual argan oil components meet therapeutic efficacy in humans as attested by the beneficial metabolic effects of argan oil reported in several clinical trials on dyslipidemic markers [11]. Improvements of dyslipidemia by argan oil are consistent with studies on the expression of nuclear genes controlling lipid metabolism which, as highlighted in the next section, have shown in an animal model of human sepsis that argan oil upregulates hepatic expression of PGC-1*α*, leading to parallel coactivations of the nuclear receptors—PPAR*α*, ERR*α*, and HNF-4*α*—which govern FAOx metabolism and gluconeogenesis [6].

## 2. Studies with Protection by Argan Oil of LPS-Induced Model of Sepsis

**Figure 1:**
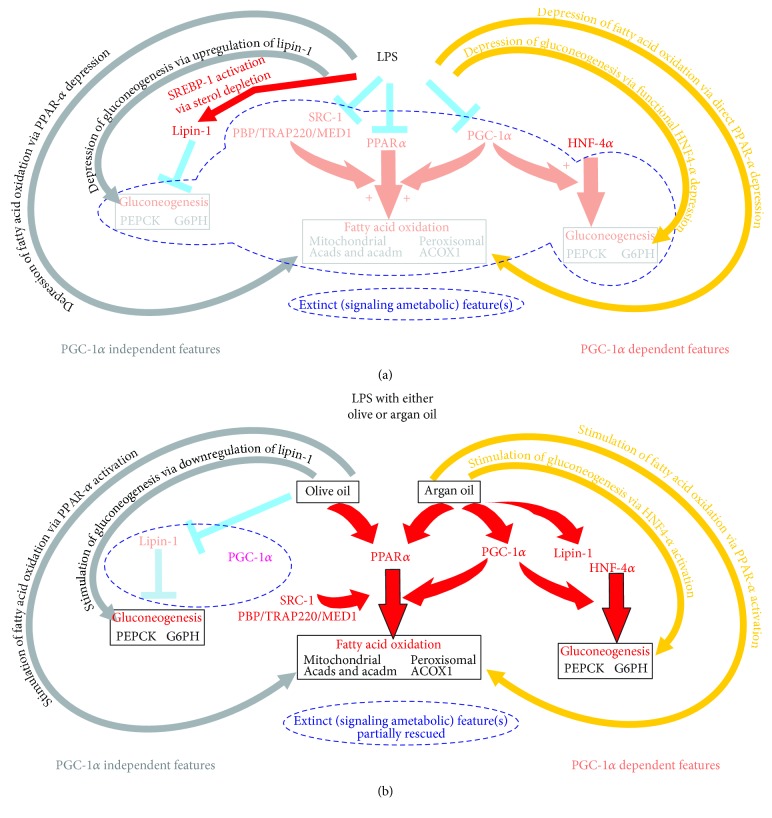
Putative impact of LPS on signaling events involved in the regulation of key steps in liver metabolic support of extrahepatic tissues (a) and effective preventive protection conveyed by argan and olive oils (b). LPS and oil effects have been divided into PGC-1*α* independent and dependent features. Note that, however, for sake of illustration, the inhibitory action of olive oil on PGC-1*α* is artificially placed in the PGC-1*α* independent mechanisms subpanel.

Chronic administration of argan oil prevents liver dysfunction experimentally induced by sepsis-mimicking conditions [6]. This result was obtained in mice exposed to LPS as a model for human sepsis. Preventive protection was observed at reasonable oil diet concentrations (6%), suggesting a potential applicability of this diet measure in the medical practice. This observation makes chronic argan oil intakes preventive antidotes for sepsis and, hence, might suggest influence of diet backgrounds of patients on sepsis development and prognosis. Whether therapeutic issues may ultimately emerge from administration of this oil, though promising, still remains speculative. Nonetheless, mechanisms by which its administration is capable of blunting sepsis LPS-driven liver dysfunction are remarkable and provide liver with a unique point-to-point reply to various signaling and metabolic targets of sepsis. The various ways altering the capacity of liver engine to fulfill energetic demands of extrahepatic tissues are subject to a suited protection through stimulation or preservation of signaling and metabolic events, which are extinct by sepsis (Figure 1). Part of features enabling LPS to disrupt signaling for liver metabolic support to extrahepatic tissues and prevention by argan oil [6] is illustrated in Figure 1 panels A and B, respectively. The depicted events have been arbitrarily divided in PGC1-*α*-dependent and independent features. LPS negatively impacts signaling for fatty acid oxidation and gluconeogenesis, the two key metabolic pathways enabling liver to aliment extrahepatic tissues in energetic substrates in periods of increased energy demands. PGC-1*α*-independent LPS features include sterol depletion by LPS leading to the LPS-dependent activation of SREBP1 [13], which in turn enhances gene expression of lipin-1 [14, 15]. The accumulation of diacylglycerol, which may involve lipin-1, leads to gluconeogenesis impairment through mTOR signaling [16] (Figure 1(a), left part). PGC-1*α*-dependent mechanisms mainly lie in a severe drop in PGC-1*α* signaling with resulting loss in PPAR*α* and HNF-4*α* signaling (Figure 1(b), right part).

**Figure 2:**
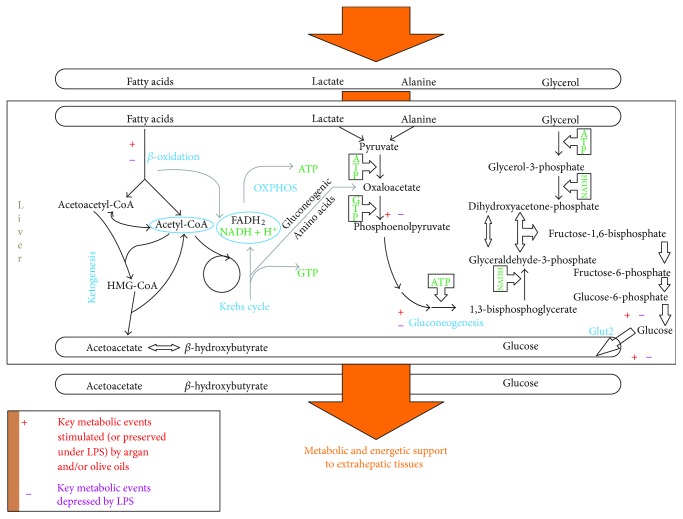
Liver metabolic and energetic support to extrahepatic tissues and its modulation by LPS, and, under LPS, argan and olive oils. Events depicted on this original figure were inferred from data published in [6].

Under LPS, argan oil (Figure 1(b), right part) stimulates PPAR*α* and PGC-1*α* signaling, contributing to stimulate fatty acid oxidation, and via the impact of PGC-1*α* acting as a coactivator of HNF-4*α*, to preserve gluconeogenesis despite no concomitant drop in lipin-1 signaling by this oil [6]. Under LPS, depression of lipin-1 by olive oil (Figure 1(b), left part) would avoid a negative impact on gluconeogenesis signaling. By contrast to argan oil, olive oil does not fully rescue the LPS-decreased PGC-1*α* expression. So, this may prevent the lowering of fatty acid oxidation activity and allows its preservation under exposure to LPS [6].  Figure 2 illustrates that argan and olive oils preserve normal hepatic fatty acid oxidation and gluconeogenesis capacities under LPS. Under LPS, each of the pathologically disrupted steps is preserved under chronic administration of the oils. As highlighted by the figure, oils may be considered as affording a protection highly adapted to the metabolically toxic liver changes induced by LPS.

## 3. Transposing to Aging the Sepsis Model of Inflammation Induced by LPS and Resolved by Argan Oil

The liver metabolic changes and underlying signaling characterizing LPS-induced model of sepsis and counteraction by argan oil have been mentioned and illustrated above. What has been illustrated in Figures 1 and 2 is essentially how the major liver energetic metabolic pathways (gluconeogenesis, mitochondrial fatty acid oxidation) and underlying signaling are modified by LPS and under LPS how argan oil may impact LPS-induced changes. Effects on peroxisomal *β*-oxidation, which is not per se an energetic pathway, have not been depicted in these figures. Interestingly enough, one of the toxic effects of LPS on mouse liver metabolism was a significant drop in acyl-CoA oxidase 1 activity [6]. LPS-mediated inhibition of peroxisomal *β*-oxidation activity was also described in rat liver under experiments focusing on the effect of endotoxin treatment on peroxisomal *β*-oxidation enzymology [17]. In this study, peroxisomal *β*-oxidation measured as lignoceric oxidation rates and ACOX1 activity was reduced to 56 and 73% control values, respectively [14]. Argan oil prevented LPS-induced drop of the ACOX1 activity in liver from mice exposed to LPS [6].

**Figure 3:**
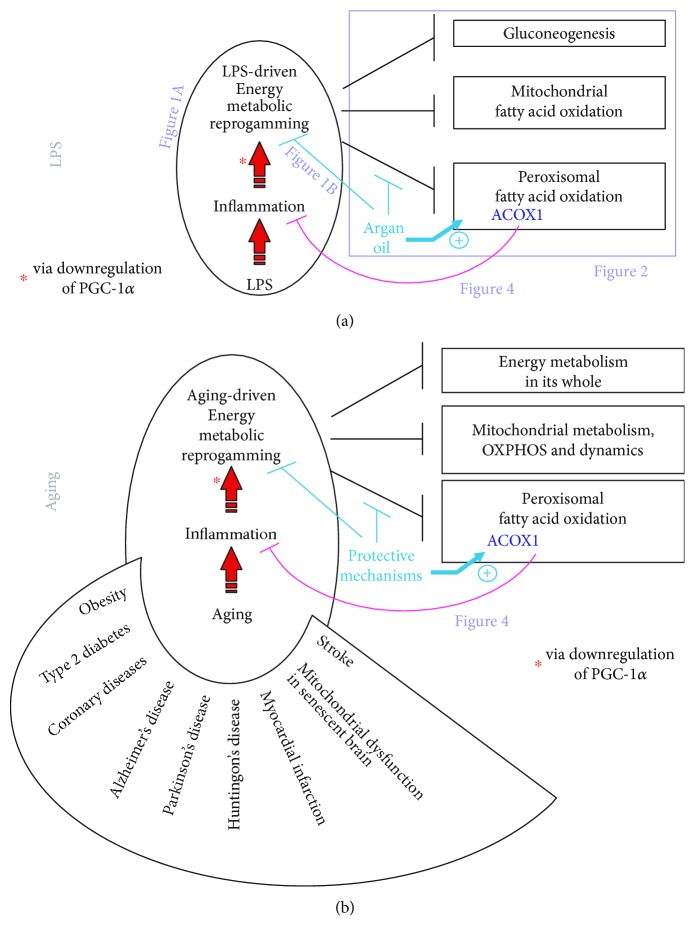
Modelizing the LPS-driven inflammation and counteraction by argan oil with a special emphasis on ACOX1 (a) and transposing this model to aging (b). For both, LPS toxicity and aging-related disorders, models, inflammatory states, and illustrated consequences along with anti-inflammatory ACOX1 and its status in these pathological states are further discussed in the text. The figure also positions other figures of this review.

Figure 3 is an attempt to model LPS-driven toxicity and protective effect of argan oil with a special emphasis on ACOX1. This representation is interesting because it attributes a key role to ACOX1 in controlling inflammation in a way upstream of the metabolic reprogramming induced by LPS. This reprogramming of energetic metabolism by LPS has been shown to involve depression of PGC-1*α* activity [10, 18]. In our representation appearing in Figure 3(a), this reprogramming of energetic metabolism is induced by inflammation and leads to impairment of ACOX1 explaining that protection (by argan oil) of this enzyme might restore an active control of inflammation and hence a unique opportunity to remove the energy metabolic reprogramming responsible for toxicity (by LPS). In our previous study on mouse model for sepsis, LPS induced a deep drop in PGC-1*α*; argan oil effectively protected ACOX1 and removed energy metabolic reprogramming as attested by full restitution of PGC-1*α* [6].

A remarkable feature is the appropriateness to transpose to aging our model of LPS-driven toxicity and protective effect of argan oil with a special emphasis on ACOX1. This is done and illustrated in Figure 3(b). Like LPS, aging promotes inflammation. It is well accepted that aging is characterized by a delay to resolve inflammation [19]. Another extraordinary parallelism between LPS and aging is the drop in PGC-1*α*, along with sometimes rescue by a rise in PGC-1*α*, which has been observed and incriminated in aging [20] and the development of many aging-related disorders including obesity and type 2 diabetes [21, 22], coronary diseases [23], neurodegenerative disorders (Alzheimer [24, 25], Parkinson [26–28], and Huntington [29–31] diseases), myocardial infarction [32–37], mitochondrial dysfunction in senescent brain [20, 38, 39], and stroke [40–43].

Consistent also with the relevance of the emerging model of aging appearing on Figure 3 are as developed thereafter *(i)* the remarkable anti-inflammatory potential of ACOX1 through generation of precursors of lipid mediators with potent anti-inflammatory activity (specialized proresolving mediators, SPM), *(ii)* protection given by either argan oil use or ACOX1 induction in signaling pathways improving diabetes and metabolic syndromes and *(iii)* protection given by SPM in age-related disorders.

## 4. Studies Highlighting that ACOX1 Is Physiologically Involved in the Synthesis of Precursors of Specialized Proresolving Mediators (SPM)

### 4.1. Peroxisomal Acyl-CoA Oxidase 1 in Health and Disease

#### 4.1.1. LPS Reduces Liver ACOX1 in a Way Counteracted by Oils

One of the toxic effects of LPS on liver metabolism was a significant drop in acyl-CoA oxidase 1 activity [6]. One of the putative protective effects of oils, argan and to a lesser extent olive oil, is prevention of LPS-induced drop in the expression of the peroxisomal protein [6].

#### 4.1.2. ACOX 1 in the Physiology of Peroxisomal *β*-Oxidation

Acyl-CoA oxidase 1 (ACOX1) belongs to one of the two separate *β*-oxidation systems identified in peroxisomes [44]. In the first peroxisomal *β*-oxidation system, ACOX1 catalyzes the first and the rate-limiting step of straight-chain fatty acids [45]. In contrast, structurally unrelated acyl-CoA oxidases 2 and 3, of the second peroxisomal *β*-oxidation system, metabolize branched-chain fatty acids (pristanic acid) and C27 bile acid precursors, respectively, although in the humans type 2 is the only acyl-CoA oxidase involved in bile acid biosynthesis, and both type 2 and 3 acyl-CoA oxidases are functional on branched-chain fatty acyl-CoA [44, 46].

#### 4.1.3. Anti-Inflammatory Potential of ACOX 1

ACOX1, one of the targets of LPS [6], has proven anti-inflammatory properties and, in the context of sepsis, its preservation by oils, under signaling adaptations abundantly developed above, might contribute to liver protection. Anti-inflammatory action of ACOX1 is based on various pathogenesis and physiological pieces of evidence considered thereafter.

*(1) ACOX1 Null Mice*. Mice lacking ACOX1 manifest severe inflammatory steatohepatitis with increased intrahepatic H_2_O_2_ levels and hepatocellular regeneration [47]. This hepatic oxidative stress may trigger TNF*α* production by Kupffer cells. Progressively, chronic endoplasmic reticulum stress contributes to hepatocarcinogenesis [48] and this steatotic ACOX1 null phenotype can be reversed by expression of the human ACOX1 [48, 49]. In the ACOX1-dependent hepatocarcinogenesis, the involvement of nuclear receptor PPAR*α* is essential [50]. In brain lesions of patients with peroxisomal *β*-oxidation deficit, oxidative, inflammatory, and apoptotic processes have been described [51]. The expressions of genes coding for cytokines IL-6, IL-8, and TNF*α*, which are typically produced by macrophages and by CD4+ T cells Th1, have also been found to be increased in ACOX1 deficiency [52]. On the other hand, several cytokines and chemokine mRNAs are strongly downregulated in ACOX1-deficient patient fibroblasts, including CXCL14 and CXCL12 genes, which have been shown to participate in the regulation of cell or tissue homeostasis [52]. In this peroxisomal disorder, lipid derivatives with an abnormally high proportion of VLCFA residues have been proposed to trigger the initial cascade of the inflammatory process [53]. The severity of the metabolic disruption associated with peroxisomal ACOX1 deficiency underlines the crucial role of peroxisomes in synthesizing or degrading highly specific metabolites, accumulation or deficit of which may impact peroxisome biogenesis itself and/or collaborative working with other cellular organelles such as mitochondria and endoplasmic reticulum.

**Figure 4:**
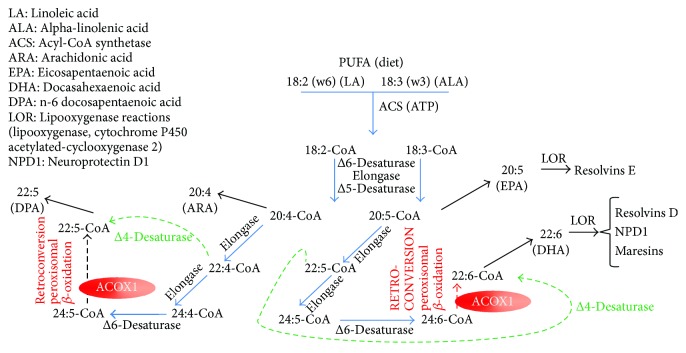
Key role of peroxisomal acyl-CoA oxidase 1 in the control of inflammation and as a target of diseases and consequent therapeutic approaches.

*(2) Physiological Role of ACOX1 in the Metabolism of Polyunsaturated Fatty Acids (PUFA)*. Physiological evidence for a key role of ACOX1 in alimenting docosanoid formation (for metabolic steps, elongases and desaturases, of PUFA synthesis considered throughout the text thereafter (see [54]) rests on the key role of this enzyme in the retroconversion of polyunsaturated fatty acids (PUFAs) (Figure 4) [7, 54–59]. For recall, families of PUFAs are characterized by the position of the unsaturation closest to the terminal, so-called *ω*, methyl of the fatty acid carbon chain. In contrast to the position of the unsaturation towards the carboxylate, that towards the other end of the carbon chain is kept upon elongation or shortening of the carbon chain. Among these families, the ω-6 and ω-3 families are generated from linoleic and *α*-linolenic acids, respectively. The metabolism of these PUFAs is illustrated on Figure 4 and starts with their preliminary activation into CoA esters by acyl-CoA synthetases prior to handling by acyl-CoA desaturases and elongases.

## 5. PUFA Desaturases and Elongases

Acyl-CoA desaturases introduce *cis*-double bonds between the carboxylate group and the first unsaturation of the carbon chain, at three carbons from this unsaturation. The desaturase(s) acting on C_18_ and C_24_ PUFAs is a Δ6-desaturase and that acting on the C_20_ PUFAs is a Δ5-desaturase. The latter desaturase produces CoA esters of arachidonic acid or ARA (5,8,11,14-eicosatetraenoic acid) and EPA (5,8,11,14,17-eicosapentaenoic acid). Nonesterified forms of PUFAs are released from phospholipids by PLA2. They are substrates for cyclooxygenases and lipooxygenases, which drive the C_20_ PUFAs (eicosanoids) towards prostanoids (prostaglandins and thromboxanes) and leukotrienes (plus HPETE, hydroperoxyeicosatetraenoic acids, and HETE, hydroxyeicosatetraenoic acids) syntheses, respectively [60–62]. CoA esters of ARA and EPA not having been incorporated into phospholipids may continue elongation.

## 6. PUFA Retroconversion

Though this view has been recently seriously challenged (see consideration below in the text), mammalian cells are assumed to possess little or no direct PUFA Δ4-desaturase activity, so the ARA-CoA and EPA-CoA elongation products in C_22_ (7,10,13,16-docosatetraenoyl-CoA [*ω*6] and 7,20,13,16,19-docosapentaenoyl-CoA [ω3]) would not be immediately desaturated at the Δ4 position. In order to produce the corresponding Δ4-desaturated metabolites, cells are capable of operating an additional elongation converting C_22_ (7,10,13,16-docosatetraenoyl-CoA [ω6] and 7,20,13,16,19-docosapentaenoyl-CoA [ω3]) to C_24_ (9,12,15,18-tetracosatetraenoyl-CoA [ω6] and 9,12,15,18,21-tetracosapentaenoyl-CoA [ω3]) products. As mentioned above, these C_24_ PUFAs are substrates for Δ6 desaturase. The C_24_ products desaturated in Δ6 (6,9,12,15,18-tetracosapentaenoyl-CoA [ω6] and 6,9,12,15,18,21-tetracosahexaenoyl-CoA [ω3]) may undergo a two-carbon chain shortening which generates C_22_ PUFAs now presenting with an unsaturation at the Δ4 position. So, cells may catalyze Δ4 desaturase activity on the C_22_ 7,10,13,16-docosatetraenoyl-CoA [ω6] and 7,20,13,16,19-docosapentaenoyl-CoA [ω3] by combining elongation in C_24_-CoA, C_24_ PUFA-CoA Δ6 desaturation and C_24_ Δ6 PUFA-CoA retroconversion into C_22_ Δ4 PUFA-CoA. This C_24_ to C_22_ retroconversion corresponds to a peroxisomal *β*-oxidizing cycle with two-carbon-chain cleavage. PUFA-CoA desaturation and elongation are catalyzed by microsomal proteins, elongation being also catalyzed by mitochondria.

### 6.1. Metabolic Intervention of ACOX1 in PUFA Retroconversion

In the scope of PUFA retroconversion, the C_24_ Δ6 PUFA-CoA requires import into peroxisomal matrix to which acyl-CoA *β*-oxidation enzymes belong. C_24_ Δ6 PUFA-CoAs are very long straight chain fatty acyl-CoAs. Transfer through peroxisomal membranes would be here assisted by ABCD2, which prefers unsaturated very long-chain fatty acids in contrast to ABCD1, the protein deficient in X-linked adrenoleukodystrophy, which preferentially acts on saturated VLCFA [63]. After import by ABCD2, the peroxisomal *β*-oxidation of C_24_ Δ6 PUFA-CoAs may proceed with peroxisomal acyl-CoA oxidase 1 (1st *β*-oxidation step), D-bifunctional protein (2nd and 3rd *β*-oxidation steps), and thiolases 1 and/or 2 (for the 4th and last step) [63]. This unique *β*-oxidation turn produces the C_22_ Δ4 PUFA-CoAs: DPA (4,7,10,13,16-docosapentaenoic acid [*ω*6]) and DHA (4,7,10,13,16,19-docosahexaenoic acid [ω3]) (DPA without other precision here refers to the n-6 DPA, the retroconversion product of TPA, and not to n-3 DPA, the product elongation of EPA).

## 7. Biosynthesis of Anti-Inflammatory Lipid Mediators from PUFAs “Retroconverted” by Peroxisomal ACOX1-Dependent *β*-Oxidation

As highlighted by Figure 4, in the absence of dietary DHA, though ACOX1 would not be needed for the formation of part of the resolvins, namely E series resolvins, it represents an essential step in the biosynthesis of the D-series resolvins, neuroprotectins, and maresins as a result of its key role in the retroconversion of PUFAs. On this light, it may be considered that the role of sepsis-mediated inhibition of ACOX1 might have been overlooked in the inflammatory challenge raised by sepsis in liver. In the same time, it may be asked whether protection of ACOX1 has not been also previously overlooked as an important feature in the anti-inflammatory potential conveyed by argan and olive oils.

The anti-inflammatory role of ACOX1 may be linked to its contribution to the yield of anti-inflammatory compounds. In this respect, DHA may be supplied by diet and endogenous biosynthesis. The last step in DHA biosynthesis is PUFA retroconversion which rests on a single peroxisomal ACOX1-initiated *β*-oxidation turn. Several works reviewed in [64] argue that resolvins, such as neuroprotectin D1 (NPD1), mediate anti-inflammatory and protective action of n-3 PUFAs in brain disorders. NPD1 is formed from DHA and reduces apoptosis in Alzheimer brain [65], whereas DHA and NPD1 lower transcript levels of the inflammatory gene products COX2 and NF-*Κ*B [66]. Protective effects of DHA and metabolites against chronic inflammations including cardiovascular disorders, asthma, rheumatoid arthritis, and inflammatory bowel diseases reviewed in [67] might also stress anti-inflammatory properties of ACOX1. In these studies, ACOX1 was bypassed by direct supplementation of its product (DHA) and derived metabolites (specialized proresolving mediators of the D series).

### 7.1. A Subtle Equilibrium between Desaturation, Elongation, and Oxidation of PUFA-CoA Esters

Desaturation and elongation of very long-chain PUFAs share in common with acyl-CoA oxidase 1 their action on CoA esters as substrates. It is likely that a subtle equilibrium exists between a given PUFA-CoA and its partition between the three fatty acid chain remodelling pathways and therefore this metabolic partition might be specific to the considered PUFA. The Δ6 desaturase involved prior the acyl-CoA oxidase 1-driven retroconversion corresponds to the fatty acid desaturase 2 (FADS2) gene product. Importantly, FADS2 is also responsible for the catalysis of a direct Δ4-desaturation of ARA and EPA directly to DPA and DHA, thus bypassing the retroconversion step (broken green arrow on Figure 4) [68] and complicating the metabolic partition mentioned above. On the other hand, in an animal model for acyl-CoA oxidase 1 gene KO, accumulation of very long-chain PUFAs were observed, corroborating the role of the peroxisomal oxidase in their retroconversion [69].

## 8. Anti-Inflammatory Potential of ACOX1 in Aging-Related Disorders Including Diabetes and Metabolic Syndrome

The recent advance in the biosynthesis of resolvins and recognition of their targets including various receptors among which GPCRs and also various microRNA (for a recent review, see [70]) has obviously heightened the interest in proteins intervening upstream formation of direct precursors of resolvins.

An important window to consider argan oil and ACOX1 as worthy regulators of inflammation perhaps lies in findings made in major disorders related to aging and known to involve inflammation. In this light, argan oil has been experimentally shown to be effective in blunting some of the features of diabetes and even in preventing chemically induced diabetes, displaying antihypertensive and antiproliferative properties, reducing in diabetic patients oxidation of LDL [71–74]. ACOX1 was found to be increased by compounds including FGF19 (Fibroblast growth factor 19, a hormonal gut-derived peptide) and nordihydroguaiaretic acid (a Creosote bush metabolite), which have been shown to improve metabolic syndrome [75, 76]. Nordihydroguaiaretic acid is endowed with many biological properties including free radical scavenging, lipooxygenase inhibition, modulation of nuclear factor erythroid 2-related factor 2 (Nfr2)/antioxidant response element (ARE) signaling pathway, and cytoprotective and toxic properties towards normal and cancerous cells, respectively; this polyphenolic compound has also proven improvement in many disease conditions such as cancer, nephropathy, and neurodegenerative disorders (for valuable reviews, the reader may be kindly referred to [77, 78]). In fact, nordihydroguaiaretic acid has been shown to counteract major events, leading to metabolic syndrome and, in this context, to protect liver metabolism, fading high-fat-diet-induced hypertriglyceridemia and hepatic steatosis ([76] and references therein). Among changes induced by the phenol compound is increased transcript expression for ACOX1, which has been considered for its metabolic oxidizing role as regards to protection given by nordihydroguaiaretic acid. However, taking into account the known role of inflammation in the development of steatosis (for a review, see [79]), protection given by nordihydroguaiaretate-induced ACOX1 against steatosis might result from anti-inflammatory properties of ACOX1. FGF19 is upregulated by farnesoid X receptor (FXR) and is physiologically involved in carbohydrate, bile, and fatty acid metabolism (for a review, see [80]). It improves glucose and lipid homeostasis and tissue response to insulin, favours weight control and energy metabolism, and as a whole has a beneficial impact in diabetes, heart and kidney diseases, and in obesity and metabolic syndrome [80]. All these aging-related disorders face both metabolic impairment and inflammation, and therefore the fact that ACOX1 is one of the target proteins upregulated by FGF19 [75], might, not only metabolically but also by anti-inflammatory mechanisms, contribute to the health benefits attributed to FGF19. Recently, however, the therapeutic potential of stimulating FGF19 and related signaling has been shaded by showing mitogenic potential conveyed by part of FGF19 protein targets [75]. Additional understanding for the mechanisms specifically mediating metabolic effects and those specifically involved in proliferative effects of FGF19 is awaited in order to uncouple these effects to meet acceptable therapeutic development.

## 9. SPM Protection in Aging-Related Disorders

Aging is long known to reduce peroxisomal *β*-oxidation and to lower ACOX1 [81]. Interestingly also, aging is associated with an inflammation status notably resulting from a less efficacy of aging to resolve acute inflammation injuries [19]. This lowered effectiveness of the anti-inflammatory response has been shown to be associated with a drop in SPM formation [19]. Accordingly, age-related decline in resolving inflammation is corrected by providing SPM or one of their important precursors, DHA [19]. In aging and in several aging-related diseases, SPM deficiency and/or disease-improvement have been documented. Aging brain is protected by DHA and DHA-derived SPM (notably neuroprotectin D1) [34, 82–85], which also improve outcome of brain ischemia-reperfusion episodes [82–85]. DHA precursors, n-3 PUFAs, and DHA-derived SPM, neuroprotectin D1, improve mitochondrial dysfunction of senescent brain [86]. In Alzheimer's disease and other degenerative disorders, DHA, SPM or SPM yield-enhancing strategies have proven efficacy in improving neuronal survival and inflammation-driven disease pathogenesis [34, 82, 83, 85, 87–94]. Age-related retinal degeneration and vision disturbances have also been found to involve disturbances in DHA and SPM and to be protected by SPM through cell survival [82, 83, 85, 87, 89, 95]. Brain ischemia/reperfusion and stroke may be improved by SPM-driven survival signaling and reduction in infiltrating leucocytes [82–84, 90]. Atherosclerosis has been also supported to be favourably modulated by SPM [96].

## 10. Anti-Inflammatory Roles of Peroxisomal ACOX1 versus Mitochondrial *β*-Oxidation

Most abundant fatty acids have a long and a straight carbon chain. When assessing the oxidation of these fatty acids as an energy-producing pathway, both mitochondrial and peroxisomal *β*-oxidation are considered, the former and the latter exhibiting major and minor contributions to overall fatty acid oxidation and the products of the latter being also potential substrates for the former. ACOX1, being the rate-limiting step in the peroxisomal oxidation of straight chain fatty acids, its activity is assayed to account for peroxisomal fatty acid oxidation capacity. Besides a limited role in energy production, ACOX1-driven peroxisomal *β*-oxidation, because taking place in the yield of potent anti-inflammatory metabolites, also plays a role in inflammation control as largely covered in this review. This role of ACOX1 remains, however, overlooked often in favor of its role in carbon-chain shortening and hence clearance of very long-chain fatty acids or in the contribution, even if minor, in energy production by ACOX1-driven peroxisomal *β*-oxidation. Whereas peroxisomal *β*-oxidation-driven protection against inflammation rests on generation of precursors for potent anti-inflammatory mediators, mitochondrial fatty acid oxidation-driven anti-inflammatory protection results from its capacity of removing proinflammatory compounds such as saturated fatty acids. These latter have been shown to promote insulin resistance, oxidative stress and oxidative damage and, in this respect, mitochondrial fatty acid oxidation meets anti-inflammatory function through avoidance of both ROS generation and lipotoxicity and through blunting of palmitate-induced proinflammatory cytokines, ER stress, and oxidative damage [97–102]. On the opposite, mitochondrial fatty acid oxidation may also promote inflammation through inflammasome activation and synthesis of the proinflammatory IL 1*β* [103], making mitochondrial fatty acid oxidation, depending on the context, both inhibitor and stimulator of inflammation. In contrast to peroxisomal ACOX1-driven *β*-oxidation, mitochondrial fatty acid oxidation would not contribute to the synthesis of precursors for SPM. However, mitochondrial VLCAD (very long chain acyl-CoA dehydrogenase) and LCAD (long chain acyl-CoA dehydrogenase) KO mice have been described to exhibit tissue decrease in DHA (docosahexaenoic acid) levels [104], and whether this effect might result from a lack of contributing or regulatory effect of mitochondrial fatty acid oxidation or in turn interfering effect of mitochondrial enzyme deficiency through accumulating metabolites on DHA biosynthesis still remains to be elucidated. To our knowledge, SPM levels have still not been reported in peroxisomal and mitochondrial *β*-oxidation disorders.

Regarding the links between mitochondrial fatty acid oxidation and aging, age-dependent decrease in mitochondrial pathway [105], as well as either increase [106] or decrease [107] in CPT1 rate-limiting step have been reported. Age-dependent decrease of mitochondrial fatty acid oxidation has been shown to be dependent on age-related glutathione drop which also enhances ROS formation and promotes insulin resistance, these metabolic and signaling alterations being corrected by cysteine-based supplementations dedicated to restore intracellular glutathione stores [105]. Increased mitochondrial fatty acid oxidation might finally improve aging as for instance attested by its ability to alleviate age-related liver lipid accumulation [108].

## 11. Conclusion

Recent works on argan oil in prooxidative and inflammatory animal/human models provide a convergent sound support for the antioxidant/anti-inflammatory role of the peroxisomal enzyme, acyl-CoA oxidase type 1 (ACOX1). The model of LPS-driven toxicity and protection by argan oil has been transposed to model similarly aging inflammation also driven by reduced PGC1-*α* activity and also considered with a special emphasis on anti-inflammatory and hence antiaging key role of ACOX1. From the one hand, this view of ACOX1 is consistent with the key role played by peroxisomal ACOX1 in the production of precursor(s) for the synthesis of lipid mediators directly involved in the resolution of the inflammation process, and for this reason called resolvins. Resolvins and related compounds such as neuroprotectins and maresins are currently referred to as specialized proresolving mediators (SPM). On the other hand, aging and aging-related disorders are associated with reduced PGC1-*α* function, may be associated with reduced ACOX1 activity, lowered SPM levels, and delayed resolution of inflammation and may be improved by strategies rising ACOX1 (metabolic syndrome and diabetes) and SPM (brain and eye aging along with corresponding neurodegenerative disorders, atherosclerosis, and stroke). The strategical metabolic place occupied by ACOX1, upstream of SPM biosynthesis, along with ability of ACOX1 preservation/induction and of SPM to improve aging-related disorders and with the fact that aging is characterized by a drop in ACOX1 and SPM, all argue towards the view that ACOX1 represents a previously unsuspected and currently emerging antiaging protein.
